# Hospitalization, mortality, and health service delivery pattern among Iranian Hajj pilgrims by age, sex, and province in 2013–22

**DOI:** 10.3389/fpubh.2025.1451591

**Published:** 2025-03-13

**Authors:** Pirhossein Kolivand, Peyman Saberian, Jalal Arabloo, Peyman Namdar, Taher Doroudi, Ali Marashi, Masoud Behzadifar, Fereshte Karimi, Soheila Rajaei, Behzad Raei, Seyed Jafar Ehsanzadeh, Arash Parvari, Samad Azari

**Affiliations:** ^1^Research Center for Emergency and Disaster Resilience, Red Crescent Society of the Islamic Republic of Iran, Tehran, Iran; ^2^Department of Health Economics, Faculty of Medicine, Shahed University, Tehran, Iran; ^3^Department of Anesthesiology, Imam Khomeini Hospital Complex, Tehran University of Medical Sciences, Tehran, Iran; ^4^Health Management and Economics Research Center, Health Management Research Institute, Iran University of Medical Sciences, Tehran, Iran; ^5^Department of Emergency Medicine, Metabolic Disease Research Center, Qazvin University of Medical Sciences, Qazvin, Iran; ^6^Iranian Red Crescent Society, Haj Medical Center, Tehran, Iran; ^7^Shefa Neuroscience Research Center, Khatam Al-Anbia Specialist Hospital, Tehran, Iran; ^8^Social Determinants of Health Research Center, Lorestan University of Medical Sciences, Khorramabad, Iran; ^9^Department of Health, Safety, and Environment Management, School of Public Health, Zanjan University of Medical Sciences, Zanjan, Iran; ^10^English Language Department, School of Health Management and Information Sciences, Iran University of Medical Sciences, Tehran, Iran; ^11^Department of Epidemiology and Biostatistics, School of Public Health, Tehran University of Medical Sciences, Tehran, Iran; ^12^Hospital Management Research Center, Health Management Research Institute, Iran University of Medical Sciences, Tehran, Iran

**Keywords:** Hajj pilgrims, mortality, hospitalization, mass-gathering, Iran

## Abstract

**Background:**

This study aims to investigate the patterns of hospitalization, mortality, and services provided to Iranian Hajj pilgrims from 2013 to 2022 by age, sex, and province.

**Methods:**

We conducted a pooled cross-sectional study in 2023, encompassing all Iranian Hajj pilgrims from 2013 to 2022. We examined pilgrim hospitalization by age, sex, and province using a Poisson regression model, incorporating demographic variables. Data extraction was performed using file reading methods, and analysis using Excel 2019 and SPSS 27 software.

**Results:**

The highest mortality among Iranian pilgrims was recorded in 2015 (*N* = 509, 74.3%), primarily affecting those aged 45–70 (*N* = 442, 64.5%). Male pilgrims experienced a greater mortality count (580, 84.7%) compared to female pilgrims (105, 18.1%). The main causes of death by province were the Mina stampede and cardiovascular diseases (CVDs). The number of hospitalizations reached its highest level in 2019 (89,492 cases) and was at its lowest in 2022 (38,947 cases). Tehran province reported the greatest number of hospitalizations (73,168 cases), while Ilam (723 cases) and Kohgiluyeh and Boyer-Ahmad provinces (868 cases) had the fewest. With the exception of heart attacks, other factors significantly impacted the average number of pilgrim hospitalizations at a 5% error level. For instance, the hospitalization figure for female pilgrims was 0.73 times that of male pilgrims, and each additional unit of pilgrimage contributed to a 0.05% increase in hospitalizations.

**Conclusions:**

Tackling the public health challenges associated with mass gatherings is crucial for protecting the health of attendees and ensuring the safety of communities. Effective strategic planning, focused interventions, and strong health surveillance systems are essential to reduce risks and build resilience for future events

## 1 Introduction

Annually, Muslims worldwide embark on the Hajj pilgrimage to Saudi Arabia. In recent years, the Hajj has seen participation from over 2 million individuals across 140 countries. Al-Hajj Al-Akbar is an annual pilgrimage to Makkah (Mecca), and Al-Hajj Al-Asghar, popularly known as Umrah, is an ongoing pilgrimage to Makkah, which takes place during all months and days of every year. In normal times, about 10 million people travel from different parts of the world to participate in these pilgrimages. The duration of Hajj Umrah pilgrims is 10 days, 5 days in Medina and 5 days in Mecca. The average number of pilgrims in the Hajj is about 33 days (1, 2).

This escalating number of pilgrims poses challenges to global health security, alongside issues related to accommodation, food, water, transportation, communication, hygiene, population control, and security. This mass gathering is confronted with health challenges such as increased disease incidence, diminished access to healthcare, disease management, and notably, emergency evacuations. The spread of infectious diseases at this gathering, coupled with the impact of chronic diseases on the mortality rate among Hajj pilgrims, has elicited concerns (3).

Given that the pilgrims are generally older than the average population and many have underlying medical conditions, their presence at this mass gathering intensifies the risk of cardiovascular diseases (CVDs), fluid and electrolyte imbalances, respiratory diseases, and other infectious diseases, including emerging diseases such as Middle East Respiratory Syndrome (MERS) and COVID-19 (4). In essence, pilgrims attending the Hajj grapple with severe communicable and non-communicable health problems. This issue contributes to the propagation of infectious diseases such as respiratory/airborne diseases, meningitis, food and waterborne diseases, and blood borne diseases (5). This congregation is anticipated to facilitate the transmission of pathogenic organisms such as meningitis, influenza, pertussis, hepatitis A, and polio. However, the most prevalent complaint among pilgrims during this journey is respiratory symptoms akin to influenza, which disrupts the performance of Hajj rituals and can even necessitate hospitalization (6, 7).

Furthermore, the prevalence of chronic diseases in Arab and Muslim countries surpasses that in non-Muslim countries. Additionally, a substantial portion of Hajj participants are over 60 years old. Given the higher prevalence of chronic diseases in the older adults, the number of individuals with chronic diseases during the Hajj can be significantly higher than estimated, leading to increased mortality and hospitalization rates (3).

Data on hospitalization and mortality patterns in healthcare facilities during Hajj can inform public health planning for this ritual and guide the optimal allocation of resources and provision of services for pilgrims. Furthermore, this data can identify gaps in the healthcare system and facilitate improved healthcare delivery. Numerous studies have reported on the patterns of diseases and mortality among pilgrims admitted to hospitals during the Hajj (3, 8, 9).

In a study conducted between 2012 and 2017, among the patients discharged from Saudi hospitals, 2,237 patients succumbed during hospitalization. In the Hajj season of 2012, 1,315 individuals passed away in Mecca and the holy sites' hospitals, and in 2017, 657 individuals died. In 2012 and 2017, 40.0% and 68.8% of these deaths, respectively, occurred in hospitals. The overall mortality rate due to all causes fluctuated over the years, with the highest rate in 2012 (42 per 100,000 pilgrims) and the lowest in 2017 (28 per 100,000 pilgrims). In the same study, <1.0% of all pilgrims were hospitalized during their stay in Mecca and the holy places (3).

The Saudi Ministry of Health provides comprehensive healthcare services to all pilgrims, including those from Iran. The ministry has established a wide network of health facilities and trained thousands of healthcare professionals to effectively manage the influx of pilgrims during the Hajj season. These services are designed to address a broad spectrum of health issues, particularly focusing on communicable diseases and chronic conditions such as cardiovascular diseases, which are common among older pilgrims. Studies have shown that a significant number of hospital admissions during Hajj are related to cardiovascular problems, underscoring the critical need for efficient healthcare delivery throughout this large-scale event (10).

The Iranian Red Crescent Society (IRCS) is responsible for conducting thorough medical evaluations for Iranian pilgrims prior to their journey to Saudi Arabia for Hajj. These evaluations follow a comprehensive protocol established by the IRCS Medical Board, which includes both clinical and para-clinical assessments to create health profiles for each pilgrim. The purpose of these profiles is to identify a range of health issues, such as cardiovascular diseases, hypertension, respiratory disorders, diabetes, and mental health conditions. Additionally, all pilgrims receive influenza and pneumococcal vaccinations at least 15 days before their departure to ensure their wellbeing during the pilgrimage. To maintain ongoing health surveillance throughout the journey, the IRCS implements a syndromic surveillance system that monitors pilgrims for 19 specific diseases. If any pilgrim experiences health issues while traveling, they receive initial assessments from physicians within their travel groups, with the option for referral to Iranian hospitals in Mecca and Medina if necessary. Caravan Physicians are equipped to perform essential laboratory tests on-site. The health data collected through these processes is crucial for identifying disease trends and informing future health strategies for Hajj pilgrims (11).

Also, Iran provides healthcare services in the Kingdom of Saudi Arabia, particularly for Iranian pilgrims during the Hajj pilgrimage. The IRCS is instrumental in delivering medical assistance to these pilgrims. It has established hospitals and clinics in key locations such as Mecca and Medina, where a variety of healthcare services are offered, including general medical care and specialized treatments. During the Hajj season, a significant number of Iranian medical personnel are deployed to ensure that pilgrims receive the necessary support and care throughout their journey (12, 13).

This study aims to describe the hospitalization, mortality, and health service delivery patterns among Iranian Hajj Pilgrims by Age, Sex, and Province in 2013–22, and also evaluate the impact of preexisting chronic diseases on hospitalization and inpatient all-cause mortality hospitals during the Hajj. It should be noted that Following the Mina disaster in 2015, Iranian pilgrims abstained from participating in the Hajj pilgrimage in 2016. Additionally, due to the COVID-19 pandemic, there were no foreign pilgrims allowed during the Hajj in 2020 and 2021. Consequently, there is no available data for those years.

## 2 Methods

### 2.1 Ethical considerations

This study is an offshoot of the research project bearing the Code of Ethics IR.RCS.REC.1402.019, sanctioned by the Iranian Red Crescent Society. The study was conducted at the Research Center for Emergency and Disaster Resilience, under the aegis of the Red Crescent Society of the Islamic Republic of Iran, Tehran, Iran. Owing to the retrospective nature of the study, data were analyzed anonymously. Consequently, the requirement for written informed consent was waived by the Research Ethics Committee of the Iranian Red Crescent Society before the commencement of the study. All stages of the study, encompassing the design, implementation, and reporting, were executed without any involvement from patients or the public. All methods were implemented according to the Helsinki guidelines and regulations.

The present study was conducted utilizing cross-sectional data collated in 2023. The study examined the pattern of mortality and hospitalization among Hajj pilgrims by sex, province, and year from 2013 to 2022. Additionally, all demographic information, risk factors, prevalence of chronic diseases, and services provided were extracted from the medical records system of the Hajj and Pilgrimage Medical Center through file reading.

### 2.2 Sampling and inclusion and exclusion criteria

No sampling was conducted in this study, and all Hajj pilgrims during the study period (from 2013 to 2022) were included in the analysis. Pilgrims whose information was not fully recorded in the databases were excluded. After the tragic Mina Stampede in 2015, Iranian pilgrims chose not to participate in the Hajj pilgrimage in 2016. Furthermore, due to the COVID-19 pandemic, foreign pilgrims were barred from attending the Hajj in both 2020 and 2021. As a result, there is a lack of data available for these specific years.

#### 2.2.1 Age categories

In this study, pilgrims were categorized into four age groups: under 15, 15–44, 45–70 years, and over 70 years.

### 2.3 Data analysis and statistical tests

The Poisson regression model was utilized to analyze the extracted data. This regression model investigates the influence of the variables of interest on the logarithm of the average frequency of pilgrim hospitalizations. Given that the majority of individuals in this study were not hospitalized, the number of zeros in the data is excessive, necessitating consideration in the model. Consequently, the proposed model employed was the zero-inflated Poisson regression model.

Furthermore, considering that hospitalization may be attributable to specific diseases and may be climate-dependent (for instance, the prevalence of colon cancer is high in Ardabil province, or the prevalence of liver cancer is high in Golestan province), this correlation was accounted for based on the provincial information in the model. In other words, the model was fitted with adjustments in the standard error of the estimators, based on the Clustered Robust approach.

In the Poisson model, regression coefficients can be interpreted in two distinct ways:

Based on the change in the logarithm of the average frequency of hospitalizations: If the variable of interest increases by one-unit (e.g., variable x_1_), while the other variables are held constant, then the logarithm of the average frequency of hospitalizations [Ln(μ)] changes by β_1_ units.

Based on the Incidence Rate Ratio (IRR): If the variable of interest increases by one unit (e.g., variable x_1_), while the other variables are held constant, then the hospitalization rate will be e ^β1^.

The Incidence Rate Ratio (IRR) is a statistical tool used in epidemiology to compare the incidence rates of a specific health event, such as a disease, between two groups. It is calculated by dividing the incidence rate in the exposed group by the incidence rate in the unexposed group, helping researchers evaluate the relative risk of an event associated with a particular exposure. An IRR of 1 indicates no difference in incidence rates between the groups, while an IRR > 1 suggests a higher risk in the exposed group, and an IRR < 1 implies a protective effect. This measure is particularly valuable in cohort studies, where individuals are tracked over time to monitor the development of new cases (14).

## 3 Results

### 3.1 Descriptive statistics

In total, the study included data on 476,506 hajj pilgrims with a mean age of 55.49 years (SD = 11.25), including 49.98% females. Of the total pilgrims, 20.90% were hypertensive, and 13.57% had DM. The highest number of pilgrims, 89,492 people, belonged to the year 2019, and 73.24% were in the group aged 45–70 years (Table 1).

**Table 1:** Characteristics of Iranian Hajj pilgrims.

**Variables**	***N* (%)**
**Sex**	
Male	238,350 (50.02%)
Female	238,156 (49.98%)
**Age (years)**	
<15	137 (0.03%)
15–45	76,078 (15.97%)
45–70	348,973 (73.24%)
More than 70	51,318 (10.77%)
**Year**	
2013	61,457 (12.90%)
2014	61,364 (12.88%)
2015	61,854 (12.98%)
2017	77,962 (16.36%)
2018	85,430 (17.93%)
2019	89,492 (18.78%)
2022	38,947 (8.17%)
**Diabetes mellitus (DM)**	
Yes	64,683 (13.57%)
No	411,823 (86.43%)
**Hypertension**	
Yes	99,566 (20.90%)
No	376,940 (79.10%)

### 3.2 The frequency of hospitalizations by year, cause and province sex

The frequency of hospitalizations of Hajj pilgrims by year is provided in the Table 2.

**Table 2:** The frequency of hospitalizations by year.

**Year**	**Frequency of hospitalization**							
	**0**	**1**	**2**	**3**	**4**	**Total pilgrims**	**Total hospitalization**	**% Of**
2013	60,883	529	40	5	0	61,457	574	0.01
	(99.07)	(0.86)	(0.07)	(0.01)	(0.00)	(100.00)		
2014	61,102	250	11	1	0	61,364	735	0.00
	(99.57)	(0.41)	(0.02)	(0.00)	(0.00)	(100.00)		
2015	61,119	672	56	6	1	61,854	735	0.01
	(98.81)	(1.09)	(0.09)	(0.01)	(0.00)	(100.00)		
2017	77,741	216	5	0	0	77,962	221	0.00
	(99.72)	(0.28)	(0.01)	(0.00)	(0.00)	(100.00)		
2018	84,724	632	63	6	5	85,430	706	0.01
	(99.17)	(0.74)	(0.07)	(0.01)	(0.01)	(100.00)		
2019	88,530	884	69	8	1	89,492	962	0.01
	(98.93)	(0.99)	(0.08)	(0.01)	(0.00)	(100.00)		
2022	38,743	193	8	3	0	38,947	204	0.01
	(99.48)	(0.50)	(0.02)	(0.01)	(0.00)	(100.00)		
**Total**	472,842	3,376	252	29	7	476,506	3,664	0.01
	(99.23)	(0.71)	(0.05)	(0.01)	(0.00)	(100.00)	7,328	0.06

In this table, it shows the frequency of hospitalization by year. It means the number of cases that were admitted only once, or twice or three or four times. Also, the number of patients who have never been hospitalized is shown as zero. For example, in 2019, 88,530 pilgrims were never hospitalized, 884 were hospitalized once. Among them, 69 pilgrims were hospitalized twice, 8 pilgrims were hospitalized three times, and only 1 pilgrim was hospitalized four times.

In Table 3, the frequency of hospitalizations by province is presented. Tehran province had the highest number of hospitalized patients, totaling 73,168, while Ilam (723) and Kohgiluyeh and Boyer Ahmad (868) provinces reported the lowest numbers of hospitalized patients.

**Table 3:** The frequency of hospitalizations by Province.

**Province**	**Number (hospitalization)**					
	**0**	**1**	**2**	**3**	**4**	**Total**
Alborz	8,026 (99)	74 (1)	7 (0)	0 (0)	0 (0)	8,107 (100)
Ardabil	4,157 (99)	45 (1)	3 (0)	0 (0)	0 (0)	4,205 (100)
Bushehr	5,456 (99)	37 (1)	5 (0)	0 (0)	0 (0)	5,498 (100)
Chaharmahal and Bakhtiari	2,509 (99)	25 (1)	2 (0)	0 (0)	0 (0)	2,536 (100)
East Azerbaijan	20,724 (99)	155 (1)	13 (0)	3 (0)	0 (0)	20,895 (100)
Fars	22,342 (99)	154 (1)	13 (0)	2 (0)	0 (0)	22,511 (100)
Gilan	3,794 (99)	24 (1)	5 (0)	0 (0)	0 (0)	3,823 (100)
Golestan	22,094 (99)	188 (1)	11 (0)	2 (0)	1 (0)	22,296 (100)
Hamadan	13,114 (99)	99 (1)	10 (0)	1 (0)	0 (0)	13,224 (100)
Hormozgan	5,701 (99)	28 (0)	2 (0)	0 (0)	0 (0)	5,731 (100)
Ilam	718 (99)	5 (1)	0 (0)	0 (0)	0 (0)	723 (100)
Isfahan	44,307 (99)	273 (1)	15 (0)	2 (0)	1 (0)	44,598 (100)
Kerman	17,717 (99)	105 (1)	3 (0)	2 (0)	0 (0)	17,827 (100)
Kermanshah	5,506 (99)	35 (1)	3 (0)	0 (0)	0 (0)	5,544 (100)
Khuzestan	24,486 (99)	255 (1)	19 (0)	5 (0)	0 (0)	24,765 (100)
Kohgiluyeh and Boyer Ahmad	859 (99)	9 (1)	0 (0)	0 (0)	0 (0)	868 (100)
Kurdistan	9,101 (99)	63 (1)	7 (0)	1 (0)	0 (0)	9,172 (100)
Lorestan	7,162 (99)	73 (1)	5 (0)	0 (0)	0 (0)	7,240 (100)
Markazi	9,352 (99)	60 (1)	6 (0)	2 (0)	0 (0)	9,420 (100)
Mazandaran	21,344	160	13	1	0	21,518
	(99)	(1)	(0)	(0)	(0)	(100)
North Khorasan	7,196	70	6	0	0	7,272
	(99)	(1)	(0)	(0)	(0)	(100)
Qazvin	6,473	45	2	0	0	6,520
	(99)	(1)	(0)	(0)	(0)	(100)
Qom	13,510	73	2	0	0	13,585
	(99)	(1)	(0)	(0)	(0)	(100)
Razavi Khorasan	58,009	365	24	2	2	58,402
	(99)	(1)	(0)	(0)	(0)	(100)
Semnan	6,628	55	7	0	0	6,690
	(99)	(1)	(0)	(0)	(0)	(100)
Sistan and Baluchestan	9,329	46	2	0	1	9,378
	(99)	(0)	(0)	(0)	(0)	(100)
South Khorasan	10,342	43	3	0	0	10,388
	(100)	(0)	(0)	(0)	(0)	(100)
Tehran	72,643	487	32	4	2	73,168
	(99)	(1)	(0)	(0)	(0)	(100)
West Azerbaijan	15,341	136	13	1	0	15,491
	(99)	(1)	(0)	(0)	(0)	(100)
Yazd	16,614	109	11	1	0	16,735
	(99)	(1)	(0)	(0)	(0)	(100)
Zanjan	8,288 (99)	80 (1)	8 (0)	0 (0)	0 (0)	8,376 (100)
Total	472,842 (99)	3376 (1)	252 (0)	29 (0)	7 (0)	476,506 (100)

### 3.3 Cause of mortality by sex

Cause of mortality by sex is provided in the Table 4. For instance, the Mina incident in 2015 accounted for the majority of male mortalities during the analyzed time frame, representing over 70% of deaths. In contrast, among women, cardiac arrest was responsible for the highest number of deaths, making up more than 40% of mortalities in the same period.

**Table 4:** Cause of mortality by sex.

**Sex**			**Frequency**	**Percent**
Men	Valid	Bone marrow failure	1	0.2
		Cancer	2	0.3
		Cardiac arrest	105	18.1
		Gastrointestinal bleeding	1	0.2
		Heart failure	1	0.2
		Kidney failure	3	0.5
		Mena	414	71.4
		Myocardial infarction	22	3.8
		Pneumonia	3	0.5
		Poisoning	1	0.2
		Shock	1	0.2
		Stroke	1	0.2
		Trauma	25	4.3
		Total	580	100.0
Woman	Valid	Cancer	2	1.9
		Cardiac arrest	46	43.8
		Mena	30	28.6
		Myocardial infarction	10	9.5
		Pneumonia	4	3.8
		Shock	1	1.0
		Stroke	5	4.8
		Trauma	7	6.7
		Total	105	100.0

In Table 5, the frequency of mortality by year is presented. The highest number of deaths occurred in 2015, with a total of 509 cases.

**Table 5:** The frequency of Mortality by year.

**Year**	**Frequency**	**Percent**	**Number of pilgrims**	**% Of total pilgrims**
2011	40	5.8	N/A	–
2012	26	3.8	N/A	–
2013	28	4.1	61,457	0.05
2014	1	0.1	61,364	0.00
2015	509	74.3	61,854	0.82
2017	49	7.2	77,962	0.06
2018	10	1.5	85,430	0.01
2019	19	2.8	89,492	0.02
2022	3	0.4	38,947	0.01
**Total**	**685**	**100**

### 3.4 Cause of mortality by age group

**Figure 1:**
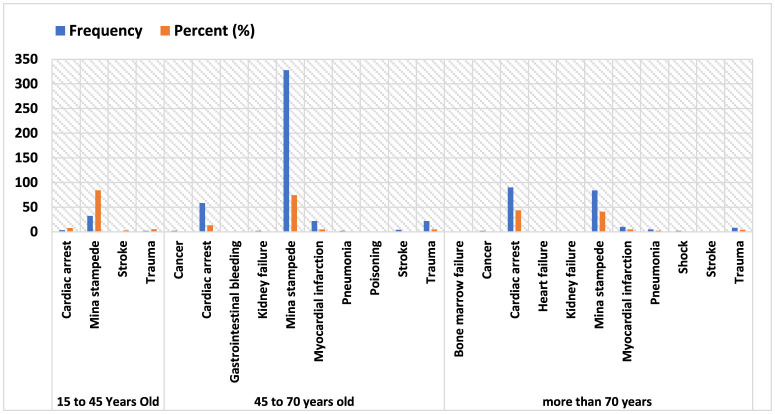
Cause of mortality by age group.

Cause of mortality by age group is provided in the Figure 1. The data presented in the table below indicates that, for the age group of 45–15 and the 70–45 years group, the Mina accident was the leading cause of death, accounting for over 80% and 70% of cases, respectively. In contrast, among individuals aged 70 and older, cardiac arrest emerged as the primary cause of death.

### 3.5 Pattern of health services for Hajj pilgrims

The Table 6 presents the number of medical services, including physician visits, nursing, medication, paraclinical and clinical services, hospitalization, and caregiving, categorized by the time from 2010 to 2022 for Hajj pilgrims.

**Table 6:** Health services for hajj pilgrims by service type and year.

**Service type (** ***N*** **)**		**2011**	**2012**	**2013**	**2014**	**2015**	**2017**	**2018**	**2019**	**2022**
Visit	Specialist	14,824 (33.3)	13,136 (24.9)	16,980 (30.3)	11,405 (32.2)	11,645 (20.1)	19,769 (20.5)	21,188 (24.2)	5,964 (16)	14,010 (10)
Nursing	Nursing services (EKG, Injection, and ...)	10,390 (23.3)	6,317 (12)	8,059 (14.4)	2,691 (7.6)	11,427 (19.7)	27,938 (28.9)	32,351 (37)	10,859 (29.2)	35,415 (25.2)
Medication	Equipment and medication	3,108 (7)	17,389 (33)	18,254 (32.6)	12,178 (34.4)	13,724 (23.7)	21,877 (22.6)	12,490 (14.3)	14,101 (37.9)	8,0195 (57.1)
Paraclinical	Laboratory tests	3,714 (8.3)	3,304 (6.3)	2,887 (5.2)	1,748 (4.9)	2,900 (5)	15,134 (15.7)	18,111 (20.7)	3,988 (10.7)	6,752 (0.1)
	Diagnostic tests	3,610 (8.1)	2,250 (4.3)	2,550 (4.6)	1,312 (3.7)	889 (1.5)	1,410 (1.5)	570 (0.7)	427 (1.1)	641 (4.8)
Clinical	Treatment interventions and surgeries	227 (0.5)	107 (0.2)	72 (0.1)	200 (0.6)	62 (0.1)	67 (0.1)	0 (0)	9 (0)	28 (0.5)
Hospitalization	General bed	483 (1.1)	324 (0.6)	344 (0.4)	216 (0.6)	396 (0.7)	806 (0.8)	440 (0.5)	56 (0.2)	40 (0)
	Special bed	221 (0.5)	216 (0.4)	224 (0.6)	117 (0.3)	106 (0.2)	166 (0.2)	67 (0.1)	27 (0.1)	67 (0)
	Emergency and outpatient	1,105 (2.5)	903 (1.7)	875 (1.6)	551 (1.6)	1,397 (2.4)	1,594 (1.6)	1,440 (1.6)	258 (0.7)	357 (0.3)
Caregiving and relief	Ambulatory care	6,874 (15.4)	8,787 (16.7)	5,786 (10.3)	4,976 (14.1)	15,432 (26.6)	7,896 (8.2)	814 (0.9)	1,545 (4.1)	2,858 (2)

### 3.6 Model specification based on the logarithmic transformation of the frequency of hospitalizations and the incidence rate ratio

This study aims to explore the impact of risk factors, in conjunction with demographic variables, on the hospitalization of pilgrims. Given that each individual may have been hospitalized more than once, the frequency of hospitalizations was considered as the dependent variable, leading to the use of a Poisson regression model, rather than merely considering hospitalization or non-hospitalization, which would necessitate the use of a logistic model.

In essence, this regression model examines the effect of the variables of interest on the logarithm of the average number of hospitalizations among the pilgrims under study. As the majority of individuals in this study were not hospitalized, the number of zeros in the data is excessive, necessitating consideration in the model. Consequently, the proposed model to be employed is the zero-inflated Poisson regression model. Furthermore, given that hospitalization may be attributable to specific diseases and may be climate-dependent, this correlation was incorporated into the model based on the information from the provinces (Tables 7, 8).

Table 7Results of Poisson regression analysis for factors associated with the frequency of hospitalization.

**Table:** 

**Hospitalization frequency**	**Coef**.	**SE**	***t*-value**	***p*-value**	**[95% Conf**.	**Interval]**	**Sig**
Female	−0.315	0.045	−7.05	0	−0.402	−0.227	^***^
Age	0.044	0.002	27.22	0	0.041	0.048	^***^
DM	0.33	0.043	7.58	0	0.244	0.415	^***^
CRF	0.894	0.118	7.59	0	0.663	1.125	^***^
HTN	0.13	0.039	3.34	0.001	0.054	0.206	^***^
COPD	0.855	0.131	6.55	0	0.599	1.111	^***^
MI	0.14	0.252	0.55	0.58	−0.355	0.634	
CVD	0.686	0.072	9.58	0	0.545	0.826	^***^
Constant	−4.893	0.12	−40.67	0	−5.129	−4.658	^***^
Constant	2.423	0.041	58.48	0	2.342	2.505	^***^

**Table:** 

Mean dependent var	0.008	SD dependent var	0.100
Number of obs	476,506	Chi-square	3084.135
Prob > chi^2^	0.000	Akaike crit. (AIC)	43758.331

^***^p < 0.01.

Table 8Results of Poisson regression analysis for factors associated with the frequency of hospitalization—Incidence Rate Ratio (IRR).

**Table:** 

**Hospitalization frequency**	**Coef**.	**SE**	***t*-value**	***p*-value**	**[95% Conf**	**Interval]**	**Sig**
Female	0.73	0.033	−7.05	0	0.669	0.797	^***^
Age	1.045	0.002	27.22	0	1.042	1.049	^***^
DM	1.39	0.06	7.58	0	1.277	1.514	^***^
CRF	2.445	0.288	7.59	0	1.941	3.08	^***^
HTN	1.138	0.044	3.34	0.001	1.055	1.228	^***^
COPD	2.352	0.307	6.55	0	1.82	3.038	^***^
MI	1.15	0.29	0.55	0.58	0.701	1.885	
CVD	1.985	0.142	9.58	0	1.725	2.284	^***^
Constant	0.007	0.001	−40.67	0	0.006	0.009	^***^
Constant (inflate)	2.423	0.041	58.48	0	2.342	2.505	^***^

**Table:** 

Mean dependent var	0.008	SD dependent var	0.100
Number of obs	476506	Chi-square	3084.135
Prob > chi2	0.000	Akaike crit. (AIC)	43758.331

^***^p < 0.01.

Our estimation reveals that, except for the variable (MI), the other variables have a statistically significant effect on the average frequency of pilgrim hospitalizations at a 5% error level. Specifically, the incidence rate ratio for hospitalization in female pilgrims compared to male ones, holding other variables constant, is 0.73 (the hospitalization rate in women is 0.73 times the hospitalization rate in men, or equivalently, the hospitalization rate in men is 1.27 times the rate in women). Additionally, for each unit increase in the age of the pilgrims, the hospitalization rate increases by 0.05%.

For the effects of other binary risk factors including diabetes (DM), chronic renal failure (CRF), hypertension (HTN), chronic obstructive pulmonary disease (COPD), and cardiovascular diseases (CVD), the interpretation is as follows: for each of the mentioned risk factors, holding other variables constant, the hospitalization rate in pilgrims with these risk factors was 1.39, 2.25, 1.14, 2.35, and 1.99 times the hospitalization rate in pilgrims without the respective risk factors.

## 4 Discussion

Mass gatherings, convened for religious, social, cultural, political, and sporting purposes, encompass a spectrum of health risks and events. Over the past decade, concerns regarding health-related risks at mass gatherings have escalated (15). According to the World Health Organization, a mass gathering is defined as a planned or spontaneous event where the large number of attendees strains the planning and response resources at the local, state, or national level (16).

Reports from most mass gatherings indicate that communicable diseases, non-communicable diseases, injuries due to overcrowding, and terrorist attacks contribute to high mortality rates among attendees. Additionally, beyond mortality, individuals may require medical care for a range of issues including endemic and seasonal diseases, dizziness, headaches, asthma and respiratory problems, limb amputations, heatstroke, and abdominal pain (17).

The mortality rate among Hajj pilgrims varies across countries, influenced by multiple factors including the health status of pilgrims, the route they take, the age distribution of pilgrims, and the presence of underlying diseases. In the present study, which examined data from 2013 to 2022, 685 deaths occurred among Iranian Hajj pilgrims. Of these, the highest mortality rate was observed in 2015, with 509 deaths primarily attributed to the Mina stampede and other factors.

The analysis of mortality rates across different age groups revealed that the highest frequency (64.5%) of mortality occurred among pilgrims aged 45–70 years old. In this context, a study on Hajj pilgrims demonstrated that the overall mortality rate during the Hajj was 216 deaths per 100,000 pilgrims. The highest mortality rate was observed among individuals over 60 years old, with most deaths resulting from cardiovascular and respiratory diseases (18).

In this study, the highest mortality rate by sex, following the Mina stampede in 2015, was due to cardiovascular diseases such as heart attacks and cardiac arrest. This finding aligns with studies conducted in other countries. For instance, a study conducted in India revealed that mortality among Indian Hajj pilgrims is primarily attributed to specific medical conditions. Among a large population of older adults individuals, cardiovascular and respiratory diseases were identified as the most common causes of mortality (19).

The mortality rate among Hajj pilgrims exhibits variation based on sex, with mortality rates generally higher in men than in women. A study analyzing the epidemiology of mortality during the Hajj reported a higher mortality rate in men (296 deaths per 100,000 pilgrims) compared to women (150 deaths per 100,000; 19). Another study conducted on Indonesian pilgrims from 2004 to 2011 revealed a mortality rate ranging from 149 to 337 per 100,000 pilgrims, with no significant difference observed between men and women (20) Consistent with these findings, the present study also reported a higher mortality rate in male pilgrims compared to female pilgrims.

An Indonesian study reported an annual mortality rate ranging from 1,765 to 3,353 per 100,000 pilgrims. The study further identified chronic kidney disease, dementia, and respiratory tuberculosis as the most common health problems causing pilgrims to postpone their travel (21). Conversely, a study conducted in Iran reported a mortality rate of 13 deaths per 100,000 Isfahan pilgrims in 1998. However, the overall mortality rate for all Iranian pilgrims was higher, with 47 deaths per 100,000 in 2004 and 24 deaths per 100,000 in 2005 (22). A study in Mecca demonstrated that mortality among international pilgrims was higher compared to domestic pilgrims, and within Mecca, it was higher than in other holy sites (3).

Hospitalization of Hajj pilgrims, attributable to various factors such as age, physical activity, and comorbidities like high blood pressure and diabetes, is prevalent and can, in certain cases, result in pilgrim deaths. In this study, the annual percentage frequency of Hajj pilgrim hospitalizations was examined. In 2019, the hospitalization rate of pilgrims was 26.26%, and in 2015, it reached its peak at 20.06%. Conversely, the lowest hospitalization rate was observed in 2022 at 5.57%. Despite the lowest hospitalization rate, due to the spread of coronavirus and other viral diseases, the utilization of medical services, including outpatient care and physician visits, and the overall burden of pilgrim visits were higher than in previous years.

In a prospective study conducted in Saudi Arabia at four hospitals in two different locations, the admission and hospitalization patterns were examined. Of the hospitalized individuals, 62% were men and 38% were women. The respiratory disorders (57%) accounted for the highest incidence of hospitalization at the hospital, followed by CVDs (19.4%) and gastrointestinal diseases (6.3%). Only three cases of hospitalization were due to heatstroke. Similarly, only one case of meningitis was confirmed in this group. In this study, underlying diseases such as diabetes, asthma, and hypertension were observed more in hospitalized individuals than other conditions (23).

In a study conducted in Iran in 2005, the most common diseases during the pilgrimage were respiratory diseases, and the incidence of these diseases in Hajj in 2005 was twice as high as in 2004. The prevalence of cardiovascular diseases in Hajj pilgrims in 2005 was 142 per 10,000, significantly lower than in 2004 (288 per 10,000). No significant differences were observed among gastrointestinal diseases, women's health, mental health, and other important diseases. Additionally, the mortality rate 2005 was significantly lower at 24 deaths per 100,000 people, compared to 47 deaths per 100,000 in 2004 (22).

In another prospective study, 545 Hajj pilgrims were examined, and demographic characteristics, health status, and disease occurrences during the journey were obtained. A significant portion of individuals had chronic medical conditions such as walking disability (26%), diabetes (21%), and high blood pressure (21%). In addition, 59% of pilgrims had at least one health problem during the pilgrimage, 44% visited a doctor during the journey and 3% of pilgrims were hospitalized. Cough was the main complaint among them (attack rate of 51%), followed by headache, heat stress, and fever (24).

In a study among Hajj pilgrims from Indonesia, a total of 72,078 people were included, 46.9% of whom were men and 53.1% were women, with the majority (35%) aged 50–59 years. In total, 42,446 pilgrims (58.9%) were classified as high-risk groups due to underlying health conditions such as high blood pressure, diabetes, or being 60 years or older. The overall hospitalization rate was 971 per 100,000 pilgrims, and the overall mortality rate was 240 deaths per 100,000 (25).

A review study was conducted to identify common health problems, such as diseases and emergencies that pilgrims face during Hajj. The analysis showed that respiratory diseases, including pneumonia, influenza, and asthma (33.73%), were the major health problems during the pilgrimage, followed by heatstroke (16.67%) and CVDs (10%). The findings also indicated that the rate of emergencies, including traffic accidents and trauma, was 3.33%. It was also mentioned in this study that 62.50% of health problems during Hajj were related to non-communicable diseases, and 37.50% were related to communicable diseases (26).

In another review article focusing on CVDs in Hajj pilgrims, CVDs have been identified as the leading cause of mortality among pilgrims. The main results of this study show that CVDs significantly contribute to mortality during Hajj compared to other communicable or non-communicable diseases. Respiratory infections are often the predominant cause of hospitalization during Hajj; however, cardiovascular diseases account for a larger portion of admissions in the Intensive Care Unit (ICU) (31).

In another study, among the discharged patients from hospitals in Saudi Arabia, 2,237 patients died during hospitalization. In the 2012 Hajj, 1,315 individuals died in Mecca and the holy sites' hospitals, and in 2017, 657 individuals died. In 2012 and 2017, 40.0% and 68.8% of these deaths occurred in hospitals, respectively. The overall mortality rate due to all causes varied over the years, with the highest rate in 2012 (42 per 100,000 pilgrims) and the lowest in 2017 (28 per 100,000 pilgrims). In the same study, <1.0% of all pilgrims were hospitalized during their stay in Mecca and the holy sites (3).

A study conducted during the Hajj in 2003 revealed that over one-third (39%) of pilgrims had underlying diseases necessitating medical care, with 22.2% having cardiovascular diseases and 19.4% being diabetic (27). Another study indicated that cardiovascular diseases were the predominant reasons for hospitalization among Hajj patients, with nearly half of emergency department (ED) visits culminating in hospital admissions (10). Data from a study on Indonesian pilgrims from 2017 to 2019 suggested that cardiovascular diseases were the second leading cause of hospitalization among Hajj pilgrims in Saudi Arabia. Among all cardiovascular cases leading to hospitalization, coronary heart disease (CHD) was the most common cause (28). In the present study, the primary cause of hospitalization among Iranian pilgrims was cardiovascular diseases (28.3%), followed by infectious diseases (16.8%) and gastrointestinal diseases (13.9%), corroborating the findings of other studies. Furthermore, an analysis of disease diagnosis patterns among all Hajj pilgrims revealed a higher prevalence of hypertension (20.2%), diabetes (13.57%), cardiovascular diseases (3.27%), and renal diseases (0.15%) compared to other conditions.

One of the results of this study was the change in the number of health services provided during the study period. One of the reasons for the change in the number of health services during Hajj is related to the change in the seasons of performing Hajj. For example, in 2015, Hajj was held in the month of September. In contrast, Hajj in 2022 took place in the month of July, this shift in timing impacts various logistical and health service arrangements due to differing weather conditions and attendance patterns (29, 30).

The study highlights several key strengths, notably its substantial sample size, which includes a diverse group of Iranian Hajj pilgrims over a decade. This extensive dataset significantly enhances the reliability and relevance of the findings, providing a strong basis for analyzing health trends within this population. The research also features a detailed demographic analysis that investigates hospitalization and mortality patterns according to age, sex, and province. This focused approach is essential for pinpointing specific at-risk groups, enabling the implementation of targeted health interventions. Furthermore, the application of robust statistical techniques, such as Poisson regression models, allows for a comprehensive assessment of factors affecting hospitalization rates while controlling for various demographic variables, thereby bolstering the credibility of the conclusions drawn.

Nonetheless, the study has some weaknesses. The methodology's dependence on file reading techniques for data extraction could lead to potential biases or inaccuracies. Moreover, the research tends to concentrate on mortality and hospitalization statistics without addressing other significant health outcomes or patient experiences that could provide a more holistic understanding of health service delivery during Hajj.

To enhance health outcomes for Iranian Hajj pilgrims, several recommendations can be proposed for health policymakers and related organizations. First, it is crucial to establish improved surveillance systems that monitor a wider array of health outcomes among pilgrims, including both communicable and non-communicable diseases, to enable timely interventions. Developing targeted health education campaigns aimed at high-risk groups identified in the study will help raise awareness about potential health risks during Hajj and promote preventive measures such as vaccinations.

Additionally, implementing mandatory pre-Hajj health screenings can assist in identifying individuals with pre-existing conditions who may be at increased risk during the pilgrimage. Encouraging collaboration among international health authorities can facilitate the sharing of best practices in managing pilgrim health. Finally, expanding research efforts to include qualitative evaluations of pilgrims' experiences with healthcare services will help identify service delivery gaps and inform policy development tailored to mass gatherings like Hajj.

## 5 Conclusion

Mass gatherings pose considerable public health challenges that require thorough planning and strategic responses. The range of health risks linked to these events—ranging from infectious diseases to injuries and potential security threats—highlights the necessity for robust health surveillance and emergency readiness.

Analysis of various studies, particularly those focused on the Hajj pilgrimage, reveals concerning mortality rates that are affected by factors like age, pre-existing health conditions, and incidents of overcrowding such as the Mina stampede. The data indicates a higher mortality risk among vulnerable populations, particularly the older adults and those with chronic illnesses, underscoring the need for targeted interventions to reduce these risks.

Continuous monitoring of health trends during mass gatherings is essential for enhancing response strategies and healthcare services. Ultimately, strengthening public health infrastructure and developing comprehensive plans will not only address immediate health issues but also build resilience for future mass gatherings. This proactive approach is crucial for ensuring the safety and wellbeing of both participants and local communities.

## Data Availability

The raw data supporting the conclusions of this article will be made available, by the corresponding author upon reasonable request.
